# Employment of PhD program graduates in Russia: a study of the University of Nizhni Novgorod graduates’ careers

**DOI:** 10.1186/s40064-015-1003-x

**Published:** 2015-05-15

**Authors:** Alexei A Mironos, Boris I Bednyi, Larisa A Ostapenko

**Affiliations:** Institute of International Relations and World History, Nizhni Novgorod State University, Ulyanov St. 2, Nizhni Novgorod, Russia; Institute of Postgraduate and Doctoral Studies, Nizhni Novgorod State University, Gagarin Avenue, 23, Building 2, Nizhni Novgorod, Russia

**Keywords:** Employment, Market of highly intellectual labor, PhD program, Surveys

## Abstract

An analysis is presented of the employment of PhD program graduates of the State University of Nizhni Novgorod, one of Russia’s leading universities, from 2009 to 2013. We have studied the features of the career paths of graduates in different disciplines who specialized in natural sciences, social sciences and humanities. The lines of research are proposed for the further study of the issues of highly intellectual labor market development in Russia and employment of PhD program graduates.

## Introduction

One of the most visible expressions of globalization processes taking place in the field of education is the Common European Higher Education Area (CEHEA) that has been formed in Europe. The changes in the higher education system are very extensive and multifaceted. They affect not only the educational systems of the leading European states, which initiated the Bologna process in 1999, but also a much wider range of states with significant specific features in the development of their national educational systems. These specific features are determined by several factors: the current level of social, economic, scientific and technological development of these countries, the traditions of national higher education systems, the goals and objectives they plan to achieve as the result of their integration in a unified European Higher Education Area.

The Russian Federation joined the Bologna Process in 2003. The analysis of the problems and results of the Bologna approaches implementation in the Russian system of higher education has been a continued focus of attention of experts engaged in research in this area (see eg, Aydarova 2014; Mingaleva and Mirskikh 2012; Vahitov 2013; Senashenko 2013; Vvedensky 2013), however, not all the problems have received equal attention. Although the formation of the ideology of a new type of doctoral education in the course of the Bologna process has been the major focus of attention over the last decade, the issues of adaptation in Russian universities of European models for organization of postgraduate training have not been adequately explored (hereinafter in this text we will consider the terms “postgraduate education”, “doctoral education”, “level 6 of Tertiary education” used in Europe and the US as synonymous to the Russian term “postgraduate studies” (“aspirantura”) and the Russian academic degree of Candidate of Sciences (“Kandidat nauk)” as an analog of the PhD degree). Currently, a number of researchers investigate individual problems of modernizing Russian PhD programs in accordance with the Bologna principles. Thus, a comparative study of the implementation of network PhD programs in Europe and Russia was carried out (Artamonova et al. 2013). Some problems encountered in the course of introduction of structured training programs and the implementation of research schools as new institutional forms of doctoral education in modern conditions in Russia were also examined (Bednyi 2013).

It is essential for all countries participating in the modernization of their education systems in accordance with the Bologna recommendations to define specific modernization measures (steps) with the account of the data relating to the employment of graduates, their satisfaction with the level and content of the training received during their period of studies. This approach is also consistent with the basic principles and objectives of the Bologna process, which include further improvement of the quality and attractiveness of European higher education and ensuring successful employment of university graduates by means of labor market orientation of all academic degrees and other qualifications.

### Problem statement

Collection and analysis of data on the employment of university graduates is a relatively new and insufficiently developed task in the framework of both the government (federal) and the departmental education statistics of the Russian Federation. A comprehensive technique for monitoring graduates’ employment developed on the initiative of the Ministry of Education and Science of the Russian Federation was tested in 2011 in 10 regions of the Russian Federation and starting from 2012 it has been used in all the constituent entities of Russia (Serova and Mazaeva 2013; Gurtov et al. 2014). However, under this system of information gathering, no information concerning employment of PhD program graduates is assembled and analyzed. Thus, at the present time there are no representative data on this subject in Russia. Such data can be obtained only in the framework of large-scale national and international studies similar to the project “Careers of Doctorate Holders” conducted by the Organisation for Economic Co-operation and Development (OECD) and Eurostat (Auriol et al. 2013) or the Survey of Doctorate Recipients conducted since 1973 by National Science Foundation (NSF), USA. (Survey of Doctorate Recipients 2013).

The objective of this study is to identify and analyze specific features of the employment of graduates of tertiary education programs (postgraduate schools) of Russian universities. This research should be viewed as an exploratory study designed to determine the most significant features of PhD program graduates’ employment and to identify the most significant problems for future research.

### Selection of the research object

In the study of employment of Russian universities’ PhD program graduates, one should take into account both the significant differences that exist between scientific and educational level of individual Russian universities, and the career paths of their graduates. There are many quite diverse factors affecting their employment prospects. Of great importance is the territorial location of the University: thus, career prospects for graduates of universities located in the capital and for graduates of provincial universities are different. This fact is not only and not so much due to the differences in the level of training, but also due to a relatively low level of territorial mobility in Russia. This also refers to highly qualified experts (Kirpichov 2011). A significant factor is the deepening differentiation between individual universities, which is stimulated by selective (competitive) support that universities receive from the government. Thus, the data on the employment of graduates of postgraduate education programs at different universities can vary significantly depending on the factors listed above. At the present stage of the study of this problem, it is important to outline the typical objects for case study that can characterize trends in the labor market for graduates of postgraduate education programs.

In this study, the analysis is based on the employment data of PhD program graduates of the Lobachevsky State University of Nizhni Novgorod (UNN). UNN is one of the major classical provincial universities that ranks at the top part of the national rankings of Russian universities. Over the last decade, it was the winner in many competitions and participated in all major government programs to stimulate the development of higher education in Russia: in 2009, UNN received the status of National Research University, it is now participating (since 2013) in the program to improve the competitiveness of Russian universities among the world’s leading research and education centers. The areas in which training is provided includes natural sciences, social sciences and the humanities. Thus, the results obtained can in a certain way (partly) characterize the employment of graduates of PhD programs of leading Russian universities in the province.

## Research methodology

To obtain information about employment, graduates who have completed postgraduate studies during the period from 2009 to 2013 were interviewed. In the course of the study, data were obtained on the “starting period” of graduates’ employment, since the respondents’ work record after graduation did not exceed 5 years. The survey involved 431 respondents (81% of the total number of graduates in this category). The distribution of respondents depending on the general areas of postgraduate studies was as follows: exact and natural sciences and IT - 62%; humanities and social sciences - 38%.

Graduates who received training in natural sciences and technology were represented as follows in respective areas of knowledge:

- physical and mathematical sciences – 48%,
- biological sciences – 22%,
- chemical sciences – 20%,
- information technology (computer sciences, mathematical modeling, informatics and computers) – 10%.

The subject-wise breakdown of graduates specializing in the humanities and social sciences was as follows:

- economics – 32%,
- law – 20%,
- history – 17%,
- political science – 16%,
- philology – 8%,
- sociology – 7%.

**Figure 1:**
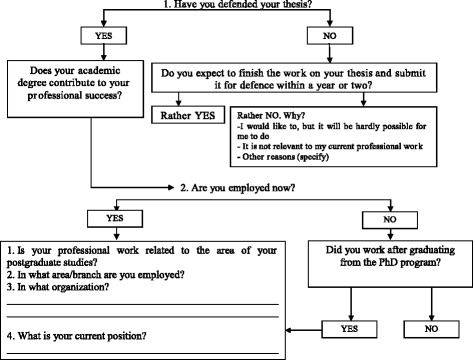
An interview script.

Interviews were conducted both during personal meetings and remotely (by phone and Skype). An interview script was developed, which can be represented as a graph: (Figure 1).

By using this technique when interviewing it was possible to get the essential information concerning employment. At the same time, the use of an extended interview (rather than a questionnaire) made it possible to ascertain some important moments in graduates’ careers and to capture some evaluative judgments expressed in the course of the conversation.

Compliance with the principles of triangulation in the study was provided by combining different methods and data sources, in particular, information obtained through interviews was supplemented by data obtained from the analysis of the University documents (databases, reports on the training in the doctoral education program).

The research was performed in the framework of Lobachevsky State University’s Program for International Competitiveness Enhancement. Informed consent was obtained from the survey participants and ethical approval was granted by the University.

## Results and discussion

There are two distinct groups of graduates in the sample that we have studied: 1 – those who have defended their theses successfully by the time of the survey and 2 – those who did not have an academic degree at the time of the survey. This is due to the fact that in the Russian system of doctoral education postgraduate training that takes 3 to 4 years and the defense of the thesis (graduation with a degree) are two independent procedures. Of those surveyed graduates who completed their training in postgraduate programs in the field of science and IT, 98.5% of respondents were employed at the time of the survey, while 44% of the respondents had a Candidate of Sciences (PhD) degree. Of the respondents in the humanities, 97% were employed, and 42% of the respondents had a Candidate of Sciences (PhD) degree. Thus, the overall employment rates between the two groups of respondents were quite close. Both in the first and in the second group, just over 40% of respondents had a Candidate of Sciences (PhD) degree at the time of the interview.

Our findings, however, suggest that along with the presence of some common haracteristics of graduates’ employment there are notable differences between the two selected groups (social sciences and humanities vs natural sciences) and between individual specialties within these blocks of disciplines.

In our study, we attempted to identify the relationship between the areas of training of PhD program graduates and the fact of their holding a PhD degree, on the one hand, and the professional fields in which they are employed, on the other. For this purpose, statistical processing of the data set obtained in the survey was carried out. We checked two hypotheses. The first one was that having (or not having) an academic degree did not affect the graduate’s choice of his/her field of professional work. Calculations were performed for each group of disciplines separately. The hypothesis was confirmed for graduates who received training in physics and mathematics, chemistry, biology, computer science, as well as for graduates in law, history, sociology and philology at the 5% significance level for the goodness of fit chi-square test. However, our data on the employment of professionals in the fields of economics and political science show a positive correlation between the graduates’ scientific degree and professional fields. The values of the association factor (above 0.8) and contingency factor (above 0.4) calculated for these two groups of disciplines indicate a high degree of correlation between the attributes considered. The second hypothesis was that the differences in the areas of studies significantly influence the choice of career paths. The testing of this hypothesis with the chi-square criterion has revealed that namely the differences in the areas of studies had the greatest influence on graduates’ career paths both for graduates with a PhD degree and for those graduates who had no such degree at the time of the survey.

### Distribution of graduates according to types of activities

Let us consider these findings in more detail. More than two-thirds of respondents with a degree in exact and natural sciences establish themselves in science and higher education (69%). It should be noted that about half of them are employed by UNN in the positions of researchers and teaching staff members. Some others are working in universities and research institutions in Nizhni Novgorod, St. Petersburg, Sarov and several other Russian cities. Some graduates (their share is less than 2%) received postdoc positions in foreign research centers and universities (Austria, Germany, Netherlands, Switzerland). These data are a good illustration of one of the most significant features of the Russian system of researcher training in postgraduate schools: the training of research and teaching personnel “for own needs” (to meet the university’s own staffing needs) remains one of the main functions of PhD programs in the majority of Russian universities. This is in contrast to Europe and North America, where the “inbreeding” in the staffing of universities is considered a negative factor.

**Figure 2:**
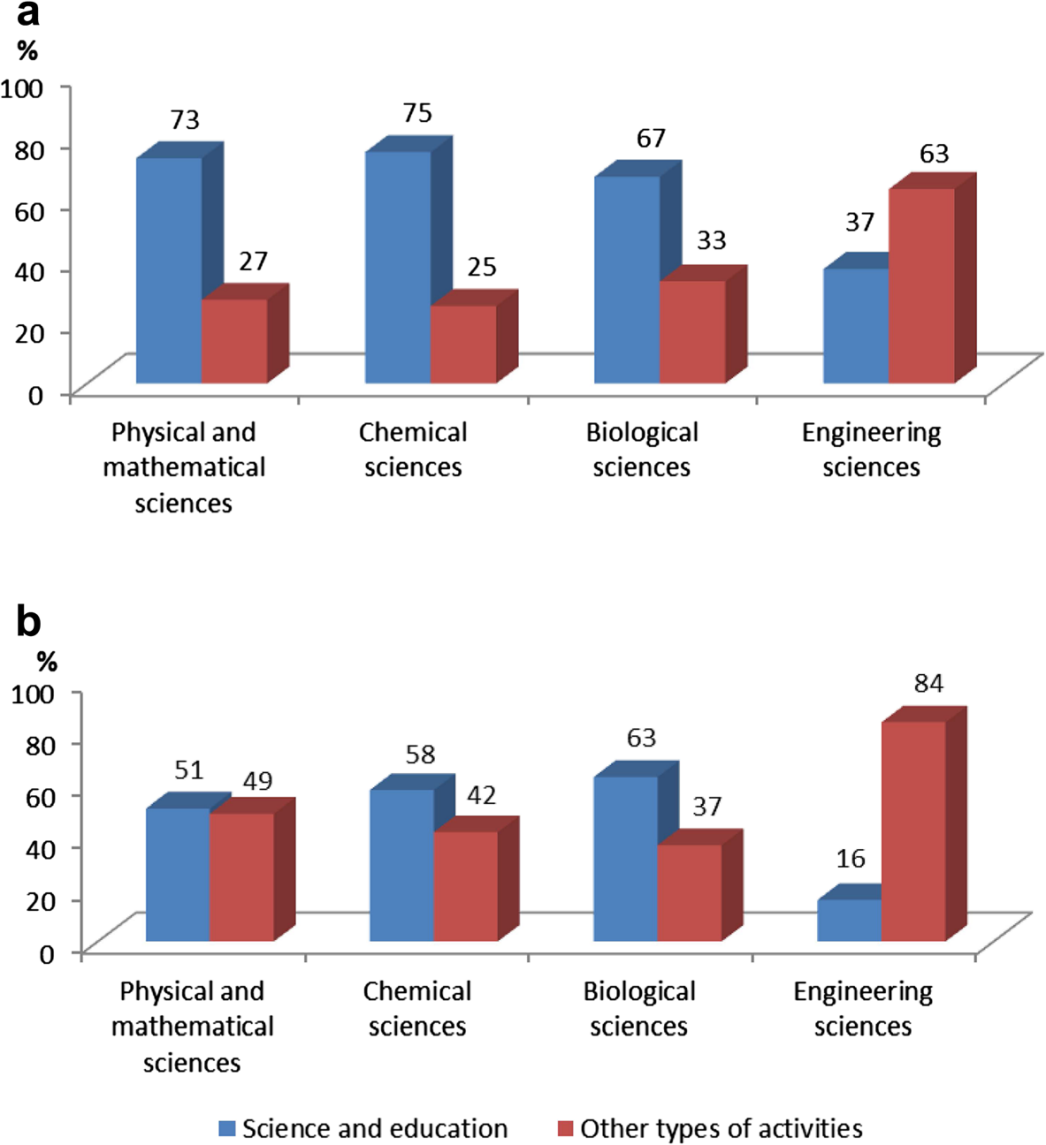
Distribution of PhD program graduates in the field of science & IT holding a PhD degree **(a)** and without such a degree **(b)**, by type of activities, %.

It follows from the data shown in Figure 2 that the research and teaching activities are most popular among those who hold a PhD degree in physical and mathematical, chemical, and biological sciences. Professionals in the field of computer science prefer higher paid practical work in the field of information technology. Interestingly, the availability or absence of an academic degree has almost no effect on career preferences of this group of graduates (cf. a & b in Figure 2). Our previous surveys of PhD students specializing in information technology (Bednyi et al. 2007) show that they are less motivated than other students to defend a thesis. Many professionals in the field of IT use postgraduate studies mainly for the development of professional and transferable skills required to obtain prestigious and high-paying jobs in high- tech companies and are little interested in obtaining an academic degree.

More than half of the graduates who were enrolled in postgraduate studies in physics, mathematics, chemistry and biology and who did not have a PhD degree at the time of the survey also chose an academic career in universities and research organizations. This choice determines their desire to complete the work on their theses and receive an academic degree (see. Figure 2b). Graduates who have not defended their thesis and are not planning to do it show a wider range of career paths.

According to the survey, 84% of graduates in natural sciences are employed in the field that corresponds to their area of PhD studies. This share amounts to 92% for those who hold a Candidate of Sciences (PhD) degree and is somewhat lower at 78% for those who graduated without a degree (note that all respondents who have received training in the field of computer science and information technology stated that their current work was in accordance with the area of training).

**Figure 3:**
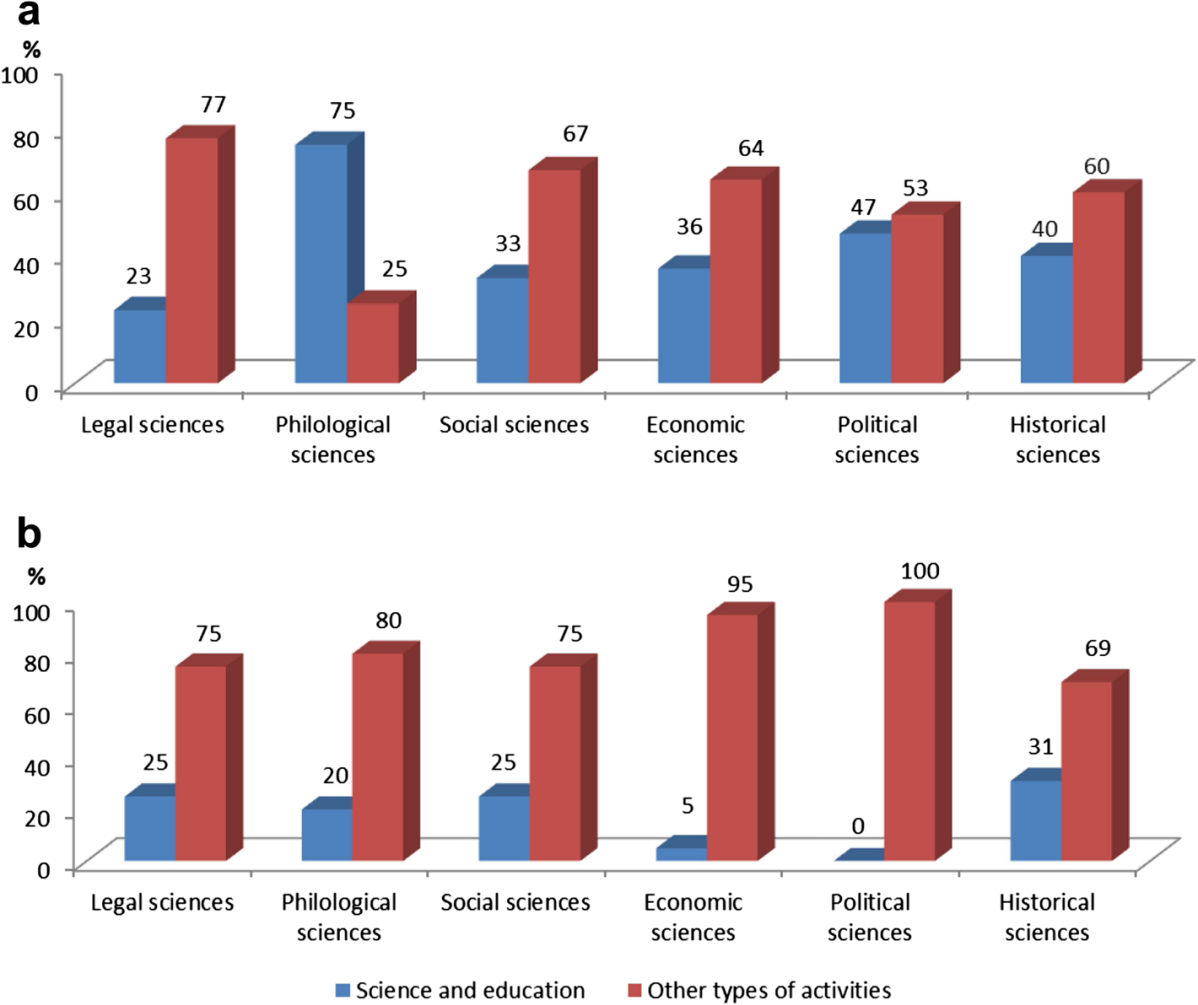
Distribution of PhD program graduates in the field of social sciences and humanities holding a PhD degree **(a)** and without such a degree **(b)**, by type of activities, %.

In contrast to PhD program graduates specializing in the area of natural sciences, the share of PhD degree holders specializing in humanities and social sciences who choose an academic career is almost 20% lower and varies from 23 to 40% for most humanities and social sciences majors. The remaining graduates (a total of more than 70%) are engaged in other activities (see Figure 3a).

From the data shown in Figure 3, significant differences can be seen in the career paths of individual groups of graduates. For professionals in the field of philology, the most important area is education and science, with 75% of graduates working there. The data obtained in the interviews show that this is not because of a particular desire of philologists to pursue an academic career. Rather, it is due as a relatively narrow range of other employment options where their skills level would be required. Scientific and pedagogical activity is of the least interest to PhDs having a degree in law, since they are in demand in a more prestigious and high-paying field of law practice. It can be seen from a comparison with the data given above in relation to the natural sciences graduates that this category of PhD program graduates is similar, in terms of their professional preferences, to graduates specializing in the field of IT, who also prefer practical activity (in their case, in IT companies).

What other professional fields are attractive to graduates of PhD programs who successfully defended their dissertations and specialized in the field of humanities and social sciences? The survey data show that there are important differences in the career paths of this category of graduates. A significant proportion of graduates (from 47 to 36% of the total number) who specialized in political science, economics and history, continue to work in higher education (see Figure 3a). Besides, attractive employment areas for degree holders in the field of sociology and history are marketing, advertising and PR (in total about 17% of graduates). To a lesser extent (about 10%) such professionals are involved in the system of state and municipal government. Economists who have a degree also find employment in the areas of commerce, consulting, logistics and in the banking sector.

The areas of professional activity of the respondents who completed their PhD programs in social sciences and humanities without defending a thesis differ significantly from the careers of those who have a PhD degree. These areas also differ from professional activity fields of graduates without a PhD degree who majored in science during their studies. Less than a quarter of graduates in social sciences and humanities without a PhD degree are employed in the field of education and science. In some groups of disciplines, such as economic and political sciences, the number of those who are employed in full accordance with the training received during their post-graduate studies is vanishingly small (see Figure 3b). Most PhD program graduates without a degree establish themselves in other areas of activity unrelated to science and higher education. These areas of activities are so diverse that it is difficult to define any preferred professional fields. Lawyers are an exception to this general rule: they prefer practice of law.

Our interviews with graduates show that 80% of PhD degree holders and 55% of graduates without a degree are employed in accordance with the areas of their postgraduate studies. The maximum number of respondents who indicated that their work was related to the area of training was among graduates of PhD programs in law and sociology.

### Professional careers of PhD program graduates

Table 1 presents the data on the professional positions occupied by PhD program graduates. A significant part of PhD program graduates in the field of science and IT holding a Candidate of Sciences (PhD) degree are employed as researchers at universities and research institutes (33%). The share of researchers is also quite high (26%) among the graduates who have completed their postgraduate studies without defending a thesis, the vast majority of them are planning to defend their theses within the next 1-2 years.

**Table 1:** **Distribution of PhD program graduates in groups of job positions, %**

**Position**	**Graduates with a PhD degree**		**Graduates without a degree**	
	**Natural sciences and IT**	**Social sciences and humanities**	**Natural sciences and IT**	**Social sciences and humanities**
Researchers	33	0	26	0
Faculty members of HEIs	28	35	7	7
Engineers and technologists	18	0	30	0
Professional staff members of companies and organizations	16	30	21	35
Management staff of companies and organizations	5	19	13	37
Other job categories that do not require high-level qualifications	0	16	3	21

The second position in the ranking of job categories of PhDs in science is occupied by university lecturers (28%). Graduates of all disciplines of natural sciences are represented here, but there are almost no specialists in the field of computer science. In the group of respondents without a PhD degree, the share of university lecturers is only 7%, and most of them also plan to defend their theses in the near future.

Thus, professional preferences of most respondents who completed their postgraduate studies in the field of science and IT are related to the research sector and to the teaching in universities. These data differ significantly from the results of a survey of PhD program graduates in social sciences and humanities. In the list of their professional occupations, researchers are missing at all, but the percentage of university lecturers among graduates in the humanities is somewhat higher than of those specializing in natural sciences (35% vs. 28%). This is due to the extremely small number of research positions related to societal and humanities issues in Russian universities and research organizations. According to our survey, in contrast to PhD program graduates in sciences, no graduates in social sciences and humanities are employed at any foreign research centers.

It can be seen from Table 1 that a significant proportion of PhDs in the field of science are involved in engineering and technological development. Most respondents in this group believe that their professional activities directly correspond to the training they received in the course of their postgraduate studies, and 67% of them believe that the academic degree is very important for their professional growth and career. Graduates of this category are working in the companies of high-tech manufacturing sector (nuclear energy, digital technology, electronic and chemical industry, shipbuilding), which readily employ young professionals with a degree in physics, mathematics and chemistry. The group of engineers and technologists ranks third among the job categories of PhDs holding a degree in science, but the same group is at the top of the list for respondents without a degree (Table 1). The respondents in this group are mainly employed in the field of IT (40%), research and educational organizations (37%) and in the manufacturing sector (14%).

As compared with graduates of humanities and social sciences programs, many of whom occupy professional or management staff positions in companies and organizations (from 49% for those with a PhD degree to 72% for the graduates without a degree), the share of graduates in the field of science holding such positions is lower (21% for PhD degree holders and 34% for those without a degree). It is in these groups of respondents that the maximum number of persons was identified whose professional activity was not related to the area of studies in their PhD program (this answer was given by 60% of PhD degree holders and by 53% of graduates without a degree holding positions of professional staff and managers). At the same time, more than half of the respondents in this group reported that their professional work required the application of knowledge and skills acquired during their postgraduate studies.

It should be noted that despite the fact that they work out of the field of their specialty, most respondents in this group stated that an academic degree was essential for their career growth. At least 74% of PhD program graduates in social sciences and humanities assessed the role of their postgraduate studies for future careers as “important”, and only 12% of philologists and 13% of graduates in the field of political science describe the role of postgraduate training in their subsequent career as “insignificant”. The results obtained in the course of the interviews are generally consistent with the data published by the Russian recruitment company “HeadHunter”. According to the published data of the survey conducted by this company among individuals with advanced degrees employed in business companies, half of them believe that an academic degree has helped them to achieve significant results in their work and career (52%). About a quarter of all respondents having a degree noted that thanks to their degree they had been able to find work in the company of their liking (23 %). Every fifth One in five received a promotion, and 18% had an increase in their salaries. Some other answers of the respondents included “greater respect on the part of other people”, “self-confidence”, “the ability to engage in teaching activities in addition to main job” (Berezina 2012).

## Conclusions

The limited scope of the study does not allow a detailed characterization of the situation of PhD program graduates in the Russian market of highly intellectual labor. However, this study makes it possible to identify some essential characteristics and features of the employment of doctoral program graduates.

The data obtained from the study demonstrate a wide range of graduates’ career paths. This confirms the need for a broad representative investigation of this problem on the national scale. A methodology should be developed for such studies along with respective metrics that would make the information obtained in the course of the studies comparable with the data of European-wide statistics on the employment of PhD program graduates.

The results obtained in the course of our study are largely consistent with those of European research on the employment of doctoral degree holders (Auriol et al. 2013). However, some significant differences are clearly visible. Thus, the fields of professional employment, in which UNN graduates specializing in natural sciences are in demand (like in most European countries), include research and higher education that ranks first, and the field of high-tech business that comes second. A significantly smaller proportion of graduates who specialized in the field of social sciences and humanities find a job corresponding to their area of training and qualifications. No more than a third of PhD degree holders work in higher education. Most graduates have to find employment in other areas. High professional mobility (readiness to take up out-of-field positions in the labor market) is combined with a very low territorial mobility of PhD program graduates.

The data obtained in the course of our research allow us to identify priority issues for further study of the employment of doctoral program graduates in Russian universities. From our point of view, it is important to undertake a more detailed study of the employment of graduates specializing in social sciences and humanities. In particular, it is necessary to examine the motives for postgraduate training in this category of students. A comparative study of the situation of this category of PhD program graduates in the labor market can provide an answer to the question whether postgraduate training in these areas is a form of “deferred employment” or postgraduate education indeed provides a significant advantage in the labor market. Special attention is required to the study of mobility problems. This includes both the mobility between different areas of activities and the trends in territorial mobility of doctoral program graduates.

The results of this study allow us to outline currently important lines of action to improve the existing PhD programs. The wide range of possible career paths of PhD program graduates calls for increased attention to the formation of transferable skills and to the development of focused programs similar to Industrial PhD Programs.
